# Acute Coronary Artery Air-Embolism after Percutaneous Lung Biopsy

**DOI:** 10.5334/jbsr.2266

**Published:** 2020-11-24

**Authors:** Flavien Grandjean, Julien Galderoux, François Cousin

**Affiliations:** 1 Radiology resident, Department of Radiology, CHU Liège, BE; 2 Department of Intensive Care, CHU Liège, BE; 3 Department of Nuclear Medicine and Oncologic Imaging, CHU Liège, BE

**Keywords:** Air emboli, Acute coronary syndrome, CT-guided lung biopsy

## Abstract

**Teaching point:** Early depiction of systemic air embolism after percutaneous lung biopsy allows for timely adequate management to prevent potentially fatal complications.

## Case Report

A 62-year-old female underwent a Computed Tomography (CT)-guided percutaneous lung biopsy for a suspicious right upper lobe nodule.

**Figure 1:**
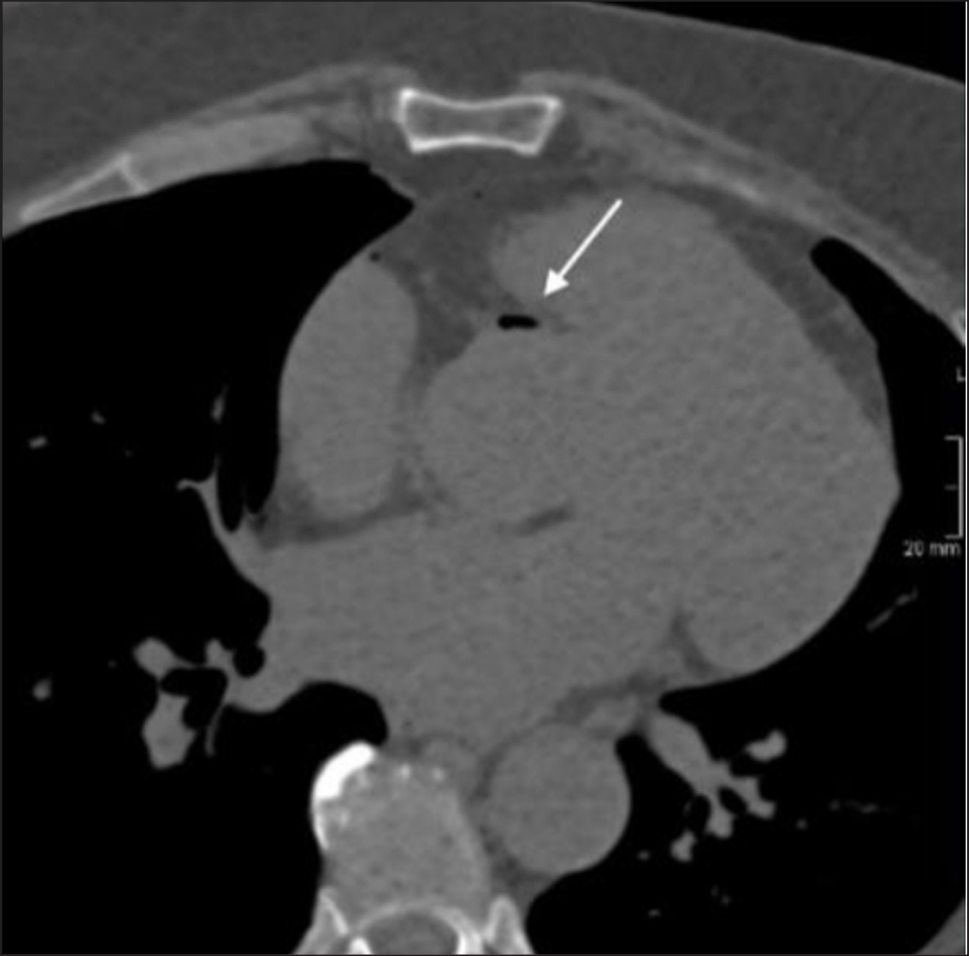


**Figure 2:**
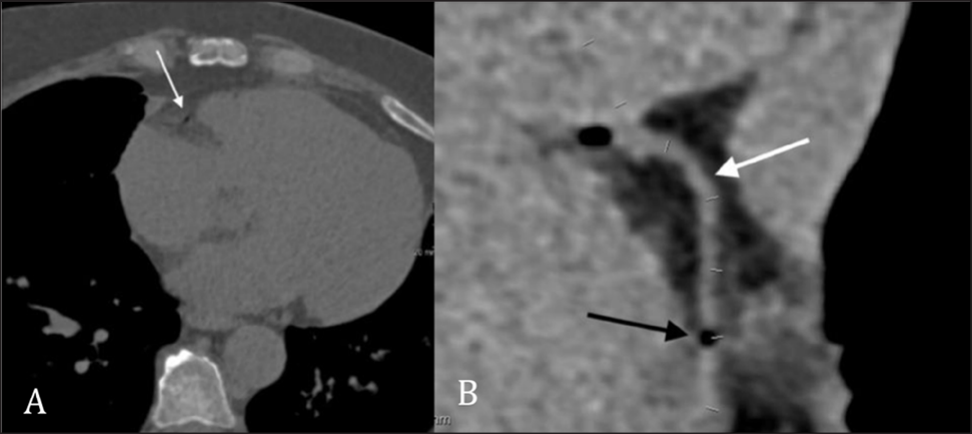


**Figure 3:**
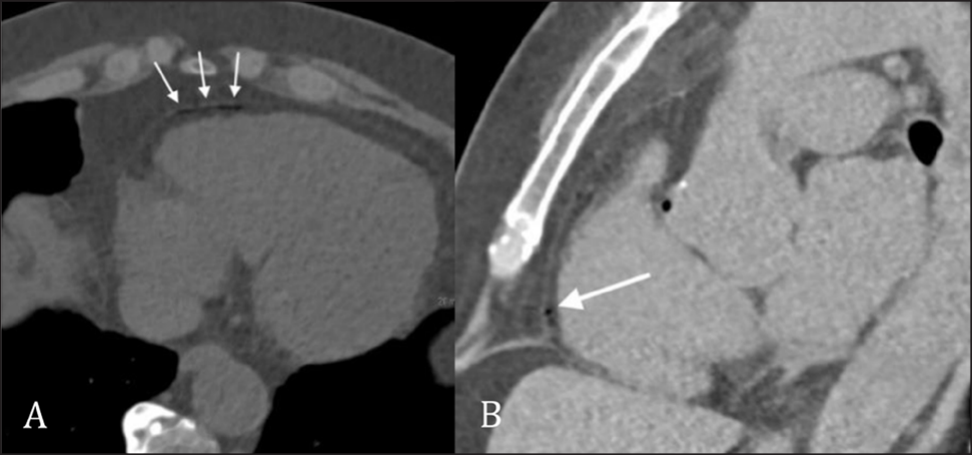


After biopsy, the patient was repositioned from prone to supine position on the CT table and presented immediately with acute thoracic pain radiating to the jaws, general discomfort and nausea. Supportive care (high flow oxygen therapy) was administered and a control chest CT depicted air emboli in the aortic bulb (Figure 1a), in the right coronary artery (Figures 2a–b) and in the right marginal artery (Figure 3a–3b). Oxygen therapy was continued and antalgic treatment was introduced. The patient was admitted to the Coronary Care Unit for close surveillance and immobilization. No myocardial ischemia was seen on echocardiography, neither cardiac enzyme elevation nor electrocardiographic modifications were seen.

Satisfying evolution followed, with disappearance of the symptoms after 2 hours. The patient was discharged the following day.

## Discussion

Percutaneous CT-guided lung biopsy is a commonly performed procedure which is relatively safe. Common complications include pneumothorax, parenchymal hemorrhage and hemoptysis, which are systematically screened for, and have a satisfying evolution when treated. Nevertheless, systemic air embolism (SAE), which is defined as the presence of gas in the systemic circulation after percutaneous lung biopsy, is not a rare complication, with an incidence of 4.8% according to *Monnin-Barres et al*. [1]. Its occurrence can have fatal consequences. Various mechanisms have been described, but the most accepted is the formation of a broncho-venous fistula (between pulmonary veins and airways) along the needle path. Significant risk factors include patient positioning (prone and right lateral decubitus) and mobilization, amount of biopsy samples and needle path length.

Patients can either be asymptomatic or symptomatic. When symptomatic, clinical signs corresponding to the affected territory are present, with cardiac and cerebral circulations being the most affected due to their high sensitivity to hypoxia.

Diagnosis of SAE is made on post-procedural CT image illustrating air emboli. It is of paramount importance to include the whole thorax in the post-procedure CT acquisition, as only focusing on the biopsy-area is proven to miss emboli. This partly explains why the incidence of SAE varies greatly between studies.

Management of asymptomatic patients resides mainly in initiating high flow oxygen therapy, monitoring cardio-respiratory parameters and prohibition of position changes. Repeated CT scans can be useful to assess air emboli disappearance. When symptomatic, the main goal is to maintain vital functions. Once stabilized, hyperbaric oxygen therapy is the first-line treatment of choice for arterial gas embolism.
